# Retroviral Elements and Their Hosts: Insertional Mutagenesis in the Mouse Germ Line

**DOI:** 10.1371/journal.pgen.0020002

**Published:** 2006-01-27

**Authors:** Irina A Maksakova, Mark T Romanish, Liane Gagnier, Catherine A Dunn, Louie N. van de Lagemaat, Dixie L Mager

## Abstract

The inbred mouse is an invaluable model for human biology and disease. Nevertheless, when considering genetic mechanisms of variation and disease, it is important to appreciate the significant differences in the spectra of spontaneous mutations that distinguish these species. While insertions of transposable elements are responsible for only ~0.1% of de novo mutations in humans, the figure is 100-fold higher in the laboratory mouse. This striking difference is largely due to the ongoing activity of mouse endogenous retroviral elements. Here we briefly review mouse endogenous retroviruses (ERVs) and their influence on gene expression, analyze mechanisms of interaction between ERVs and the host cell, and summarize the variety of mutations caused by ERV insertions. The prevalence of mouse ERV activity indicates that the genome of the laboratory mouse is presently behind in the “arms race” against invasion.

## Introduction

The activity of transposable elements (TEs) places a variable mutational load upon their host species [1–3]. In species such as *Drosophila,* TEs comprise approximately 10% of heterochromatic [4] and only 2%–3% of euchromatic DNA [4,5] but cause over 50% of de novo mutations [6]. In contrast, nearly half of the human genome is TE-derived but de novo disease-causing insertions are rare [7,8]. TE activity in the laboratory mouse falls in the middle of these two extremes [8,9], largely because of the activity of endogenous retroviruses (ERVs) and other elements with long terminal repeats (LTRs), which together make up 8%–10% of the genome [7,9–12] (Box 1). A striking difference between the mouse and the human repertoire of ERVs/LTR elements is that the mouse contains many “active” LTR retroelements and a few potentially infectious ERVs that are closely related to exogenous mouse retroviruses [9,13]. Unlike in inbred mice, infectious human ERVs have not been described, no new insertions have been found, and there are no ERVs closely related to human exogenous retroviruses [10,11,14]. In addition to LTR elements, the major classes of retrotransposons in mammals are the non-autonomous short interspersed elements (SINEs) and the autonomous long interspersed elements (LINEs) [7,9,12]. The retrotransposition and genomic effects of these non-LTR retroelements have been extensively discussed in a number of recent reviews [2,15,16].

Since the mouse is widely used as a disease model, it is important to understand the mutagenic events affecting this species and how they differ from those in humans. This article examines mouse ERVs and other LTR retroelements, focusing on insertional mutagenesis of the germ line. For the purposes of this review, LTR retroelements, which amplify via intracellular retrotransposition, and true exogenous retroviruses, which amplify by extracellular infections and retrotransposition, will be considered together as “ERVs” as they have a common evolutionary origin [13]. We discuss mutational mechanisms of different families of ERVs, illustrating significant differences in their effects on genes. We also discuss host responses to curtail ERV activity and, in some cases, to adopt ERVs for normal cell functions. Finally, we present the view that inbred mice are in a transitory state in which ERVs are not at equilibrium with their host genome.

## Prevalence of ERV-Induced Mouse Insertional Mutations

Present-day activities are markedly different between human and mouse ERVs. A recent review lists 48 TE insertion mutations in human [17], and estimates of the frequency of novel human TE insertions range from one retrotransposition per 8–33 births [2,18–20], all of them due to L1-mediated non-LTR retrotransposons (reviewed in [21,22]). Given that ~47,000 mutant alleles have been characterized (according to the Human Gene Mutation Database [http://www.hgmd.cf.ac.uk/]; August 2005), these numbers suggest that ~0.1% of human spontaneous mutations are due to TE insertions, but none are due to ERV activity. As in human, a small number of de novo germ line L1 insertions have been reported in mice (reviewed in [22,23]). However, the ongoing activity of ERVs accounts for the majority of new insertional mutations in the mouse. To provide a current estimate of the fraction of spontaneous mutations due to ERV insertion, we tabulated all documented cases and found 63 (Table S1). The Mouse Genome Informatics database (http://www.informatics.jax.org/) lists 1,489 spontaneous mutant alleles (as of August 2005). After removing unannotated cases and numerous non-independent entries and revertants derived from the *nonagouti a* allele (Box 2), 519 spontaneous alleles with an annotated molecular mechanism remained. This list included 55 of our 63 ERV insertion mutations. Taken at face value, these figures suggest that 10%–12% of all mutations are due to ERV insertions, a fraction very similar to previously reported estimates of 10%–15% based on lower numbers [22,23]. Reversion of ERV-induced mutations has also been observed at a few loci due to LTR–LTR recombination (Box 2). It should be noted that the 10%–15% figure is likely an underestimate because of ascertainment bias. For example, point mutations in coding regions will be more readily detected than ERV insertions in introns or outside gene borders. Regardless of the precise figure, ERV activity in inbred mice is dramatically higher than in modern humans.

Most ERVs are highly transcribed during early zygotic divisions and in germ cells, resulting in an increased likelihood of new heritable proviral integrations (Box 3). Although genomic copy numbers of murine leukemia virus (MLV) are low (see Box 1), this family is the most active mouse ERV on a per provirus basis. New MLV provirus acquisitions are found in 2%–75% of the progeny in the highly susceptible SWR/J-RF/J hybrid mice [24–26]. AKR mice appear to gain one new ecotropic MLV provirus every 50–100 generations [13]. Five germ line mutations or strain variants due to insertions of MLV have been well characterized in other lab strains (Table S1). The somatic effects of MLV, as well as mouse mammary tumor virus, in activating oncogenes via insertional mutagenesis are well known [27]. Indeed, mapping common retroviral integration sites in mouse tumor systems has proven a powerful strategy to identify new genes involved in cancer [28,29].

Intracisternal A particle (IAP) and Early Transposon (ETn)/MusD elements are present in much higher copy numbers than MLVs (Box 1) and are responsible for the majority of ERV-induced de novo germ line mutations (Tables 1 and Table S1). In addition, IAP elements are frequent insertional mutagens in somatic cells, particularly in leukemia, plasmocytoma, and myeloma cell lines, and can activate oncogenes or cytokine genes [30]. Notably, very few new ETn insertions have been reported in somatic cells [31], likely reflecting their restricted expression pattern or limited expression of coding-competent MusD elements, required for ETn retrotransposition.

**Table 1:**
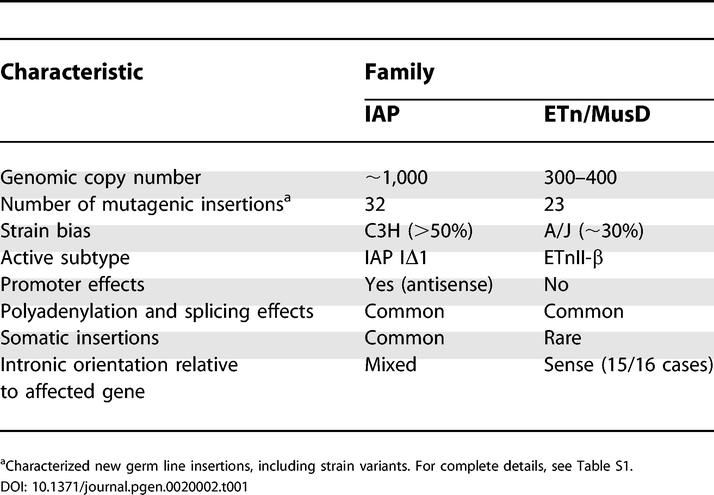
Overview of IAP and ETn Mutagenic Germ Line Insertions

## Genetic Background and ERV Subtype Influence on Insertion Probability

Not all mouse strains are equally susceptible to ERV insertions. Most of the IAP insertions have occurred in C3H/HeJ (Tables 1 and Table S1), and nearly all of these cases are of the IAP subtype IΔ1 [32,33]. It seems likely that one or a small number of IΔ1 IAP elements are active in this strain, possibly because of a favorable genomic context or escape from host suppression mechanisms (see below). The IΔ1 subtype, however, requires complementation in *trans* from coding-competent IAP elements [34], so the latter must also be expressed. A specific strain bias for ETn insertions is not as obvious, although six mutations have occurred in A/J mice and two in each of two other infrequently used strains (SELH/Bc and MRL/MpJ) (Table S1). As with IAP elements, this suggests that some strains harbor more “active” elements and/or allow more ETn or MusD expression. Where sufficient sequence is available, it has been found that most ETn insertions are of a particular structural subtype, ETnII-β, and are nearly identical, suggesting very few currently active elements ([35]; unpublished data). A very definite strain bias has been observed for germ line movement of MLVs, with AKR and SWR/J-RF/J mice being highly active strains (reviewed in [13]) (see Box 3). One of the explanations for such selective activity in particular strains may be the presence of a single highly transcribed master element in a favorable genomic context, as appears to be the case for AKR mice. The other explanation is the difference in host suppression factors, such as methylation levels and the presence of virus-suppressing loci. One long-studied locus involved in suppression of MLV and a number of other ERVs is *Fv1* (see below).

As mentioned above, most de novo insertions of IAP and ETn elements are those of defective sequences lacking full coding potential. This fact is curious, given that, in assay systems, coding-competent IAP and MusD elements retrotranspose much more efficiently when proteins and retrotransposing RNA are encoded by the same template (*cis* preference) [34,36]. Expression patterns of defective and full-length IAP elements vary widely in different cells [30,32,37], but the IΔ1 deleted subtype is preferentially expressed in acute myeloid leukemia cell lines derived from C3H/HeJ mice, despite being present at lower genomic numbers than full-length forms [32]. Non-coding ETnII elements are transcribed at a much higher level than their coding-competent MusD relatives [35], probably explaining their higher likelihood to retrotranspose. Indeed, among the 23 characterized mutagenic ETn/MusD insertions, only two have been reported as MusD (Table S1). However, it is unclear why transcripts from defective elements would predominate in vivo. Possibly they are less likely to be recognized as retroviral elements and to be repressed by host cell silencing machinery.

## Mutagenic Mechanisms of ERV Insertions

**Figure 1:**
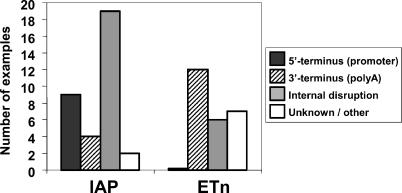
Mutagenic Mechanisms of IAP and ETn Insertions IAP and ETn insertions were classified by their mechanism of gene disruption. Well documented instances of aberrant transcription initiation (5'-terminus) and polyadenylation (3'-terminus) were counted, as well as aberrant splicing and exon skipping (internal disruption). Insertions that cause gene disruption by multiple mechanisms (Table S1) were counted once in each relevant class.

**Figure 2:**
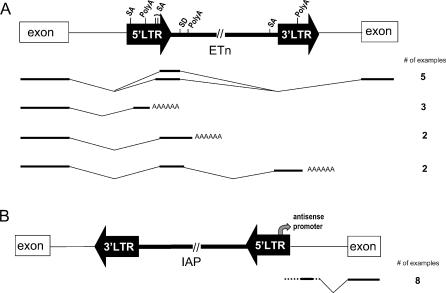
Common Effects of ETn and IAP Insertions on Gene Expression (A) ETn effects on gene transcript processing. The most common patterns of aberrant transcript processing caused by ETns in gene introns are shown. The natural LTR polyadenylation (polyA) site and a second cryptic polyadenylation site in the internal region, along with four cryptic splice acceptors (SA) and a donor site (SD), are involved in most cases. The number of such cases is an underestimate, since several reports lack sufficient detail of aberrant transcripts. In some cases, several aberrant forms have been found. Boxes denote gene exons, thin lines denote introns, and thick lines denote spliced mRNAs, with direction of transcription from left to right. For clarity, cryptic splice acceptor sites in the 3' LTR are not shown since no documented splicing events involving these sites were found. Intronic mutagenic ETns and the affected gene are most often found in the same orientation (15 of 16 cases). (B) IAP promoter effects on gene transcription. Ectopic gene expression driven by an antisense promoter in the 5' LTR of an IAP has been reported in eight cases. In some cases, the IAP is located a significant distance upstream of the gene.

Most commonly, germ line mutations due to ERV insertions occur in an intron, disrupting gene expression by causing premature polyadenylation, aberrant splicing, or ectopic transcription driven by the ERV LTR (Tables 1 and Table S1). In some cases, small amounts of normal gene transcripts and protein can still be detected. While the number of characterized MLV-induced mutations is too small to perceive general trends, IAP and ETn elements show significant differences in their effects on genes (Figure 1). For ETn insertions, the most commonly reported defect is premature polyadenylation within the ETn, coupled with aberrant splicing due to a few commonly used cryptic splice signals (Figures 1 and 2A). IAP insertions within introns also typically cause aberrant splicing but use a wider variety of cryptic splice signals. In addition, compared to ETns, fewer cases of premature polyadenylation within IAP elements are well documented (Figure 1), but, in many cases, all aberrant gene transcripts have not been well characterized.

A striking difference between the effects of IAP and ETn elements is their tendency to drive ectopic gene expression (Figure 1). For IAP elements, nine cases of LTR-driven gene expression have been reported. Interestingly, eight of these nine cases are driven from an antisense promoter located in the 5' IAP LTR (Figure 2B; Tables 1 and Table S1). Many of the mutant alleles caused by IAP LTR-driven gene expression show variable expressivity among genetically identical mice and have therefore been termed metastable epialleles [38]. The variable expressivity is due to stochastic establishment of the methylation state of the 5' LTR. If the LTR is mostly methylated, its promoter is inactive and little or no effect on the gene is observed. However, if the LTR is unmethylated, its promoter drives ectopic gene expression, resulting in the mutant phenotype. Such cases have been extensively studied by Whitelaw and coworkers, who have proposed the intriguing theory that phenotypic variation in mammals could in part be due to incomplete and variable silencing of retrotransposons in somatic cells [39].

It is unclear why no instances of ETn-promoted ectopic gene expression have been observed, but the lack of such cases could be explained by inactivity of the ETn LTR promoter in somatic cells due to heavy methylation or lack of necessary transcription factors. Expression studies (Box 3) indicate that at least some IAP elements are transcribed in various cell types, a property that would increase the probability of such elements providing promoter function. It is possible that the presence of the cryptic antisense promoter in the IAP LTR also increases the likelihood that an IAP element 5' of a gene will provide promoter function (Figure 2B).

## Host Silencing Mechanisms

### Transcriptional gene silencing.

**Figure 3:**
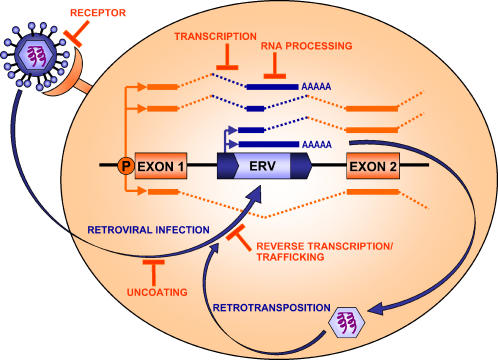
Host Restriction and Silencing of ERVs/LTRs Blocks to various stages of the retroviral or LTR retroelement life cycle are depicted as are silencing mechanisms affecting activity of integrated elements. Examples of restriction genes and silencing mechanisms: receptor block, *Fv4;* uncoating block, *Trim5;* reverse transcription/trafficking block, *APOBEC3* and *Fv1;* transcription block, CpG methylation; and RNA processing block, *Nxf1* and RNAi. See text and Table 2 for more details and other examples. An ERV or LTR element within an intron is shown to illustrate common gene-disruptive effects of such sequences through introduction of polyadenylation sites, promoters, and splice donor and acceptor sites. Spliced RNA is depicted with dashed lines. A normal gene transcript driven by the native promoter (P) is shown below the gene. A full-length retroviral transcript, which could be packaged for further rounds of retrotransposition or retroviral infection, is shown above the gene locus. Various potential aberrant or chimeric transcripts are shown above.

To guard against harmful genomic consequences of ERVs and other TEs, an arsenal of cellular defense strategies has evolved to counteract their amplification (Figure 3; Table 2). Transcriptional gene silencing is a principle mechanism for controlling TEs in a broad range of species including mammals, flowering plants, and those fungi whose genomes contain m^5^C [40]. The best-documented mechanism, DNA methylation of promoters, can directly impede access of transcription factors or lead to an inactive form of chromatin at target loci [41]. Indeed, a majority of genomic CpG dinucleotides and 5-methyl cytosines reside within ERVs and other retroelements in mammals [42]. Several lines of evidence confirm that genomic hypomethylation and TE activation are interrelated. DNA methyltransferase (Dnmt) mutant mice with mutant *Dnmt1* or *Dnmt3* do not maintain and initiate methylation at existing or new proviral loci, respectively [43,44]. In fact, both MLVs and IAPs become substantially demethylated [43], and IAP transcripts are expressed up to 100-fold higher in *Dnmt1^−/-^* mice relative to wild-type [45]. While *Dnmt1* is necessary after DNA replication, *Dnmt3a* and *Dnmt3b* are essential in the germ line and during development to establish the methylation repertoire [44]. This activity is largely restricted to dispersed and tandem repeats [46]. *Dnmt3a* and *Dnmt3b* knockout embryonic stem (ES) cells are unable to establish methylation at new MLV integrations. Knockout ES cells and embryos exhibit a general decrease in methylation at centromere repeats, MLVs, IAPs, and L1s [44]. In addition to the research on promoter methylation, there is a study showing that intragenic methylation reduces the elongation efficiency of RNA polymerase II [47], which suggests that the methylated state of TEs within introns might affect gene expression.

**Table 2:**
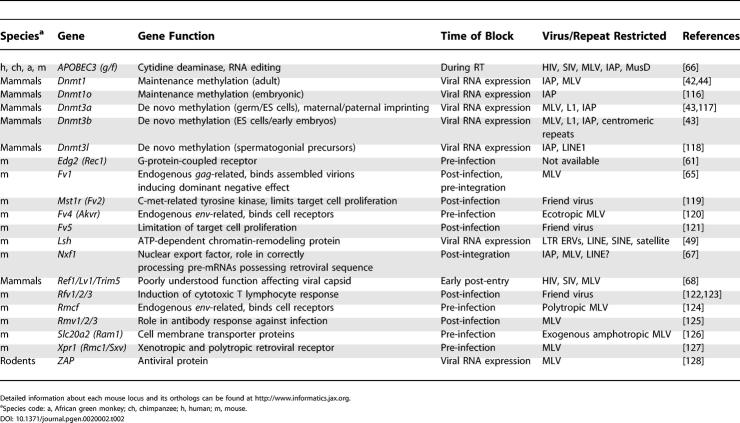
Mammalian Restriction Genes against Retroviral Activity

It is well established that genomic methylation can serve to recruit chromatin-remodeling proteins [41]. The SWI/SNF family members are components of the trithorax group protein complex and are responsible for maintaining transcriptional activity. A SWI/SNF mammalian catalytic subunit, Brm (SWI/SNF-related, matrix associated, actin-dependent regulator of chromatin), is involved in increased transcription of retroviral RNA, but this is alleviated in cells lacking this protein [48]. Moreover, Brm-deficient cells treated with histone deacetylase inhibitors are unable to silence transcription of retroviral genes. These results suggest that Brm-type SWI/SNF is essential for TE expression and that histone deacetylation is crucial for silencing. Paradoxically, Lsh (lymphoid-specific helicase), also a SWI/SNF family member, preferentially associates with repeats and contributes to their silencing [49]. *Lsh^−/-^* mice are hyperacetylated at histones overlying TEs (class I and II LTRs, LINEs, SINEs, and centromeric repeats), and their transcripts are abundant. This defect appears specific to repetitive sequences. A further level of silencing is mediated by histone methylation. Intriguingly, different families of repeats were found to have characteristic repressive histone methylation patterns [50]. Furthermore, histone methyltransferase knockout ES cells exhibited a loss of these repressive marks and an increase in transcription from tandem and interspersed repeats.

### Post-transcriptional gene silencing.

Since transcriptional silencing is unlikely to prevent activity of all TEs, it is essential that some processes act at the level of expressed transcripts. An RNA interference (RNAi)–mediated mechanism, the components of which are discussed elsewhere [51], is involved in post-transcriptional gene silencing of repetitive DNA. High levels of sense and antisense IAP and ERV-L transcripts are expressed concurrently in developing mice, but are not detected past the eight-cell stage [52,53]. Moreover, inhibiting the RNAi pathway in preimplantation embryos by RNAi-mediated knockdown of Dicer results in a 50% increase in IAP and ERV-L transcripts [52,53]. Dicer knockout mouse ES cells exhibit increased transcription from centromeric repeats, L1s, and IAPs, combined with severe developmental defects [54]. In an analogous example, silencing of the mammalian X chromosome is dependent upon an antisense transcript and shortly after its detection, histone 3–lysine 9 and CpG methylation is established at *Xist* [55], connecting double-stranded RNAs to transcriptional gene silencing. The fact that heterochromatin can be established at homologous loci via short interfering RNAs (siRNAs) is well documented. Examples in model organisms such as fission yeast and *Arabidopsis* have implicated repeat-derived siRNAs in directing such conformational changes. Fission yeast deleted for RNAi pathway components express centromeric-repeat and integrated transgene transcripts, normally heavily silenced by heterochromatinization [56]. Studies in plants show that TEs and tandem repeats specifically become silenced by histone 3-lysine 9 and CpG methylation. These changes are dependent on the chromatin remodeling factor Decrease in DNA Methylation 1 (DDM1) and guided by siRNAs. Indeed, various *Arabidopsis* genes become subject to RNAi-mediated silencing because of TE proximity to their promoters [57]. Similar results in human cells have demonstrated that non TE-derived siRNAs targeted to the *EF1A* promoter of a proviral green fluorescent protein reporter inhibits transcription of the transfected *EF1A* promoter, as well as that of the endogenous copy [58]. Also, siRNAs targeting the E-cadherin promoter induced DNA methylation and heterochromatin [59], but DNA methylation is not a prerequisite, as shown with the *CDH1* promoter [60].

### Host restriction factors.

Finally, a variety of gene products, some derived from domesticated viral genes, function at various stages of the retroviral life cycle to curtail both exogenous retroviruses and ERVs and have been extensively reviewed recently [61–64] (Table 2). Some particularly relevant examples include Fv1, the Ref/Lv1 family of proteins, APOBEC3G, and Nxf1. *Fv1,* the “prototypic” retrovirus restriction gene, is an ancient ERV-L *gag*-like gene that restricts infection by MLV [65]. *APOBEC3G* encodes a cytidine deaminase that mediates cysteine–uracil transitions when co-packaged with retroviral genomes. It inhibits HIV and MLV replication and also suppresses IAP and MusD/ETn retrotransposition [66]. *Nxf1,* encoding an mRNA nuclear export factor, has been shown to suppress the hypomorphic effects of intronic IAP insertions, presumably by facilitating accurate splicing [67]. The recently described Ref1/Lv1 family of proteins, including TRIM5α, suppresses HIV and MLV [68,69]. However, effects of these proteins on ERVs are unknown.

## Inbred Mice—Out of Balance with Their ERVs?

The evolution of silencing mechanisms by the host likely, in turn, places pressure on TEs/ERVs to evolve means to escape repression, setting up an “arms race,” not unlike that involving the immune system and infectious agents [1,70,71]. In the case of TEs, waves of amplification are countered by host defenses (Figure 3) and negative selection that quench activity until new variants or “master” elements appear that are capable of instigating further genomic expansions [1,3]. The high rate of ERV germ line and somatic insertional mutations in the laboratory mouse indicates that at least some inbred strains are currently in an active phase of ERV genomic expansion. In contrast, ERV-like elements in humans, while present in comparable overall numbers, have long ago ceased activity [7]. It is interesting to speculate which is the more common situation in modern-day mammals. Without detailed analysis of a variety of mammalian genomes and mutational spectra, it is difficult to answer this question. In mouse, the still active ETn and IAP elements likely amplify via intracellular retrotransposition, thereby avoiding the “front line” defense mechanisms, such as *Fv1* and *Fv4,* in place to inhibit early stages of exogenous infections. In contrast, MLV likely amplifies primarily through rounds of infection of germ line cells, allowing more opportunities for the host to evolve resistance and keep proviral copy number low. We propose that inbred mice represent a relatively transitory state in which host silencing mechanisms have not yet adapted to retrotransposition of new ERV variants. The IAP family nicely illustrates this point. The IΔ1 partly deleted subtype is currently the most active IAP element but is a minor fraction of the total number of existing IAPs. This situation suggests that full-length IAPs amplified to high copy numbers during mouse evolution but have recently been essentially silenced. The IΔ1 subtype must have arisen recently and, possibly because of specifics of its structure and/or genomic context, has been freed from suppression and allowed to retrotranspose—mainly in the C3H/HeJ strain. A similar scenario is occurring with respect to ETn/MusD elements, where a minor population of ETnII-β elements is causing the bulk of current retrotranspositional activity. This relatively permissive phase of ERV expansion that is ongoing in inbred mice provides a rare opportunity to study how a mammalian host genome responds to new waves of invasion by mobile elements.

## Conclusion

This review has attempted to highlight the mutational impact that ERVs have had and continue to have on the mouse germ line and to discuss host defenses that have evolved to control these elements. Unlike in human, ERVs in the mouse genome are in an expansion phase, with specific IAP and ETn variants currently playing the dominant role. These elements have accumulated to hundreds of copies in the genome, but evidence indicates that only a few have a high probability of retrotransposing. Identification of their genomic location and/or chromatin state may provide insight into host control mechanisms and why particular elements escape suppression. Genetic factors responsible for variable retrotransposition rates in different strains also await discovery and may reveal new host restriction genes or alleles. Given the propensity for these ERVs to affect gene expression, it would be interesting to investigate ERV insertions as mediators of phenotypic differences among inbred strains. Indeed, it may be particularly informative to examine genes harboring polymorphic ERV insertions in their introns.

The epigenetic control of mouse and human ERVs is of substantial interest because of their potential effects on adjacent genes. In addition to obvious gene-disruptive effects, mammalian ERVs may also play a role in tissue-specific gene regulation (see Box 4). Some IAP elements act as metastable epialleles [38] with their methylation state determining effects on neighboring genes. The idea that variable silencing of retrotransposons could contribute to gene expression variability in mammals [39] is attractive but, thus far, IAPs are the only type of retroelement shown to display this effect, and it remains to be determined how widespread this phenomenon may be. Functions for RNA-mediated silencing, including potential roles for RNAi [53] and microRNAs [72] in controlling ERVs and exogenous retroviruses, are rapidly being elucidated. A number of questions, however, including the origins of double-stranded RNA necessary for inducing silencing, are currently unanswered [73].

Although ERV insertions are not a source of new mutations in humans, understanding their effects in mice is important for understanding gene regulatory effects of existing human ERVs/LTRs, thousands of which are located within gene borders [74,75], and in elucidating the disruptive effects of therapeutic retroviral vectors. Retroviral activation of proto-oncogenes has occurred in gene therapy trials, raising major concerns [76]. Therefore, potential long-range promoter or enhancer effects, as displayed by IAP elements, need to be considered and vectors designed to reduce the chances of oncogene activation [77]. The high probability of some retroviruses integrating into introns [78,79] may also limit their usefulness as therapeutic gene delivery systems if aberrant gene splicing and polyadenylation results. Eliminating cryptic splicing and polyadenylation signals within retroviral vectors may be a worthwhile strategy. However, as demonstrated by the mouse ERVs, unique properties and sequence motifs result in distinct mutational mechanisms (see Figure 1), indicating the challenge of attempting to predict a priori the mutagenic behavior of different classes of retroviruses.

## Supporting Information

Table S1 Germ Line Mouse Mutations Caused by ERV Insertions (233 KB DOC)

## Abbreviations

Dnmt: DNA methyltransferase
ERV: endogenous retrovirus
ES: embryonic stem
ETn: early transposon
IAP: intracisternal A particle
LINE: long interspersed element
LTR: long terminal repeat
MLV: murine leukemia virus
RNAi: RNA interference
SINE: short interspersed element
siRNA: short interfering RNA
TE: transposable element

## Notes

Box 1. Classification of Mouse ERVs A plethora of ERV families exist in the mouse and are grouped into three major classes (class I, II, and III) [9,13,80]. ERVs with infectious counterparts, namely MLV and mouse mammary tumor virus, have been studied for decades and are the subject of many excellent reviews [13,81,82]. Here we focus on the less well appreciated class II IAP and ETn elements, which account for the majority of characterized germ line insertional mutations, and briefly touch on representatives of other classes. Class I retroviruses. The class I/type C/gammaretroviruses, composing about 0.7% of the genome [9] and grouped based on similarity to MLV [13], were first isolated from lymphomas of AKR mice [83]. MLV entered the germ line of mice approximately 1.5 million years ago and its copy number ranges from 25 to 70 depending on the mouse strain. MLV proviruses are subdivided based on their host ranges determined by their *env* genes, but only a few encode replication-competent viruses [13,84]. Class I also includes several other families, but it is unclear if any have fully coding-competent members [13]. Class II retroviruses. The prototype of the much more numerous class II/types B and D/betaretroviral group, composing about 3% of the genome [9], is mouse mammary tumor virus [13]. A wide variety of other mouse betaretroviral ERV families also exist, some of which retain coding capacity and appear to have entered the genome quite recently [80,85]. One of the most extensively studied noninfectious families of ERVs in the mouse is the IAP family. Though there is variation in copy numbers between different mouse strains, about 700 full-length and 300 partially deleted elements are present in the haploid mouse genome. Type I elements encompass full-length members as well as four deleted classes [37]. Of these, the IΔ1 subclass, which has a 1.9-kb deletion in *gag-pol,* is the most abundant deleted form in the mouse genome and is also responsible for the majority of IAP insertional mutations [32,33]. Type II elements differ from type I elements by a characteristic 500-bp length difference and only comprise partially deleted members [37]. IAP elements were thought to lack an *env* gene until about 200 *env*-containing elements were discovered [86]. The other major family of active mouse LTR elements are ETns, first described as a family of non-coding sequences transcribed during early embryogenesis [87]. ETn RNA levels are significantly elevated and restricted to certain tissues during embryonic days 3.5 [87] to 13.5 [88]. Two major subtypes of ETn elements, I and II, differ in the 3' portion of the LTR and a 5' internal segment. As with IAP elements, copy numbers likely vary between strains, but the February 2002 release of the C57BL/6 genome has ~200 ETnI and ~40 ETnII elements [35]. Although ETnI elements are more numerous, ETnII elements are currently more active [31]. The lack of coding potential in ETn elements raised questions as to how they could retrotranspose, but it is now clear that a related coding-competent family of endogenous betaretroviruses, termed MusD [89], that share nearly identical LTRs with ETns, provide the proteins necessary for ETn retrotransposition [36]. No MusD element has an *env*-related sequence, suggesting amplification exclusively via retrotransposition. Class III retroviruses. Mouse class III elements, which may include some active ERVs, consist of the MuERV-L family, which encompasses up to 200 proviral copies of about 7.5 kb per haploid genome [90] and different subgroups of the highly repetitive non-autonomous ORR1 and MT MaLR elements [91]. Class III elements compose approximately 5.4% of the genome [9], significantly contribute to the early mouse transcriptome [52], and affect gene regulation (Box 4). However, only one mutation due to insertion of a class III MaLR element has been documented (Table S1). Many mouse and human ERVs and solitary LTRs remain essentially unstudied, except for their annotation in the Repbase database of repetitive sequences [92]. Since such annotations are often based on incomplete information, they should be viewed with caution [85].

Box 2. Reversion of ERV-Induced Mutations In a few instances, reversion to a wild-type phenotype occurs among mice carrying ERV insertional mutations. The mechanism of reversion involves deletion of internal ERV sequences via homologous recombination between the 5' and the 3' LTRs of the provirus in the parental germ line, leaving behind a solitary LTR. Such generation of solitary LTRs has been noted in early studies on MLV [93], and this mechanism has been efficient throughout evolution. Indeed, solitary LTRs are typically present in much higher copy numbers than full proviral forms and make up the bulk of retroviral material in the mouse and human genomes [9,11]. In the case of the *hairless* mutation [94] and the *dilute* coat color mutation [95], both of which are caused by aberrant splicing due to an MLV integration into the intron, reversion to wild-type occurs via generation of a solitary LTR. Germ line reversions of the *dilute* mutation occur at a frequency of 3.9–4.5 x10^−6^ events per gamete[96], and one somatic revertant, chimeric for about 50% of reverted cells, was encountered, with the frequency of somatic reversion estimated at 9 × 10^−7^ per animal analyzed [96]. Occasionally, reversions can result in diverse phenotypes because of expression of different forms of transcripts. Such is the case with the *nonagouti a* allele, which encompasses an insertion of a 5.5-kb VL30 element containing 5.5 kb of additional internal sequence flanked by 526-bp direct repeats. By means of homologous recombination, *a* can revert to two dominant agouti alleles, *black-and-tan (a[t]),* containing only the VL30 element with a single internal 526-bp repeat, and the *white-bellied agouti (Aw),* which only has a solitary VL30 LTR [97]. There is no published evidence of other ERV-induced mutations reverting to wild-type, perhaps because of the difficulties associated with breeding the mutant animals, the time span required to detect reversions, and, for the somatic revertants, restricted tissues where the reversion could be detected.

Box 3. Dynamics of ERV Expression and Transcriptional Restriction Acquisition of new heritable proviruses requires expression in the germ line and, indeed, transcription of ERVs in different species is elevated in germ line cells, early embryo, and placenta compared to adult or differentiated tissues [98]. Active transcription during early developmental stages is advantageous to ERVs, since it increases the probability of proviral integrations likely to contribute to the germ line and be inherited by the next generation. High transcriptional activity of ERVs in early embryogenesis is partly due to lower levels of suppressive methylation, but expression profiles do not coincide exactly with the patterns of global genomic de- and remethylation [99] and are likely also the result of changing transcription factor repertoire during development. In AKR mice, a high-leukemic mouse strain, MLV transcripts are detected at high levels in the embryo and throughout the life of the animal, likely originating from one ancestral provirus in this strain [13]. In low-leukemic mouse strains, such as BALB/c, C3H/He, and C57BL/6, MLV proviruses are transcribed at significantly lower or undetectable levels (reviewed in [82]). Transcript levels of ETn and IAP elements are also highest during early embryogenesis [37,87,88,98]. While IAP elements are expressed in many mouse tumors and cell lines [37], expression of ETns is more restricted, elevated only in undifferentiated embryonic carcinoma and ES cells, as well as in primary acute myeloid leukemia cells [100]. Though IAP transcripts are detectable in some normal adult tissues and cell types, such as thymus and activated splenic B cells (for review see [37]), a reporter gene system in transgenic mice found IAP promoter activity to be restricted to undifferentiated spermatogonia [101]. This finding suggests that IAP transcripts produced in differentiated somatic tissues or tumor cells may initiate from only a very limited number of elements in favorable genomic contexts or may be influenced by other genes. Indeed, in most studies on ERV expression, it is unclear how many individual elements contribute to the transcript pool, a fact that limits firm conclusions on transcriptional activity of these large families. Time-specific restrictions placed on ERV transcription, limited largely to a narrow window of early embryogenesis, are suggestive of extremely tight regulation imposed by complex mechanisms of the host genome in an effort to prevent somatic insertional mutagenesis.

Box 4. Adoption of ERVs to Serve the Host Although the vast majority of TE and ERV insertions are selectively neutral, allowing them to drift to fixation, or detrimental and subject to negative selection, such elements can occasionally be co-opted by the host to serve important cellular functions [2,3,71,102]. For example, as mentioned in the text, endogenous viral loci can play a role in repelling exogenous retroviral infections (Table 2). Ancient ERVs may also have been co-opted to function in placental development in humans [103,104] and mice [105], prompting the suggestion that expression of different ERVs is partly responsible for the great diversity of mammalian placental structures [106]. A growing number of studies have shown that LTR elements are particularly well suited to donation of enhancers or promoters and, if fixed, can assume roles in gene regulation. Many examples of mammalian genes regulated by ERVs/LTRs or other TEs have been reported [71,74,107–110], such as the mouse *Slp (sex-limited protein)* gene, which has acquired male-specific expression due to a MuRRS ERV that provides an enhancer [111], and the *CYP19* gene, encoding a key enzyme in estrogen biosynthesis, which has high expression in human and primate placenta due to an alternative promoter provided by an ancient LTR [74]. A recent study documenting ERVs present in chimpanzee but not human found that several such elements were associated with genes differentially expressed in the two species [112], raising the possibility that ERVs and other TEs may be critical in driving speciation, as has been discussed by others [2,3,71,102]. Most ERV LTRs seem to be extremely powerful, ready-made promoters active during early embryogenesis and in the germ line, and recent work has fueled speculation that LTR retroelements could play a role in expression of genes essential for early development. Knowles and co-workers reported that about 13% of cDNAs from full-grown mouse oocytes contained retroviral sequences, the majority of which were derived from the poorly understood class III MT MaLR family [52], but by the two-cell stage, transcripts of another class III family, MuERV-L, started to predominate [52,113]. Notably, MT, MuERV-L, and some other LTRs were found to act as alternative promoters for subsets of host genes in full-grown oocytes and cleavage-stage embryos, apparently controlling synchronous, developmentally regulated expression of these genes [52]. An independent study reported that blocking MuERV-L expression inhibits embryonic development to the four-cell stage [113]. Such findings have prompted the proposal that differential gene expression driven by LTRs may trigger sequential reprogramming and genome remodeling during embryonic development [52]. This idea is intriguing, but since LTR retrotransposon families and insertion patterns are generally not conserved across divergent species, it is difficult to envisage a scenario in which such elements evolved to play a critical role in common developmental processes. It is easier to imagine LTRs/TEs being involved in species-specific processes. Nevertheless, it is clear that McClintock's original theory of TEs as “controlling elements” [114] and Britten and Davidson's postulation of repetitive elements as regulatory units [115], views that have been little appreciated for decades, are now gaining increasing attention.
