# Supplementary material for: Retroviral Elements and Their Hosts: Insertional Mutagenesis in the Mouse Germ Line
Source: PLoS Genet. 2006 Jan 27;2(1):e2. doi: 10.1371/journal.pgen.0020002 (PMC1331978; doi:10.1371/journal.pgen.0020002)
Supplement: Supplementary file 1 [file pgen.0020002.st001.doc]

# Supplementary Table 1: Germ line mouse mutations caused by ERV insertions

| **IAP insertions:** | | | | | |
| --- | --- | --- | --- | --- | --- |
| **Mutation** | **Strain** | **Location &**  **Orientation** | **Mutational mechanism(s)** | **Refs** | |
|  |  |  |  |  | |
| *A^hvy*^* | C3H/HeJ | Exon 1C,-^b^ | IAP forms 5’-end of transcript^¥^, Regulation by methylation | [1] | |
| *A^iapy*^* | C57BL/6N | Intron, - | IAP forms 5’-end of transcript^¥^, Regulation by methylation | [2] | |
| *A^iy^* | C3H/HeJ | Intron 1, - | IAP forms 5’-end of transcript^¥^, transcript contains internal IAP sequence | [3] | |
| *A^vy*^* | C3H/HeJ | Exon 1A, - | IAP forms 5’-end of transcript^¥^, Regulation by methylation | [3,4] | |
| *Ap3d^mh2J^* | C3H/HeJ | Intron 21, +^b^ | Transcript contains internal IAP sequence^‡^,Truncated protein | [5] | |
| *Atcay^ji-hes^* | C3H/HeJ | Intron 1, ?^b^ | Transcript contains internal IAP sequence^‡^ | [6] | |
| *Atrn^mg^* | C3H – Swiss stock cross | Intron 26, - | Transcript contains internal IAP sequence^‡^, Truncated protein, Likely termination in IAP | [7] | |
| *Atrn^mg-L^* | C3H/HeJ | Intron 27, + | Transcript contains internal IAP sequence^‡^, Reduced protein levels, Likely termination in IAP | [7] | |
| *Axin^Fu*^* | 129/Rr | Intron 6, - | IAP forms 5’-end of transcript, Regulation by methylation, and transcript contains internal IAP sequence^‡^, Truncated protein | [8,9] | |
| *Axin^kb*^* | 129/Rr | Exon 7, - | IAP forms 5’-end of transcript, transcript contains internal IAP sequence, Internally deleted protein | [8] | |
| *Dab1^scm^* | Dc/Le | Intron, - | Transcript contains internal IAP sequence^‡^, Truncated protein | [10,11] | |
| *Eya1^bor^* | C3H/HeJ | Intron 7, + | Transcript contains internal IAP sequence^‡^, Reduced mRNA levels | [12] | |
| *Gus^mps2J^* | C3H/HeOuJ | Intron 8, +, | Reduced mRNA size and level, No enzyme activity detected | [13] | |
| *Hps^coa6J^* | C3H/HeJ | Exon 10, - | Transcript contains internal IAP sequence | [14] | |
| *Hps^ep^* | C3HeB/FeJ | 3’-coding exon, - | Transcript contains internal IAP sequence, Protein contains IAP-encoded amino acids | [15] | |
| *Hps^ru6J^* | C57BL/6 | Unknown, + | Kidney: loss of expression, Brain: transcript contains IAP sequence | [16] | |
| *Lama2^Pas^* | Non-inbred Agouti | Intron^#^, + | Transcript contains internal IAP sequence, Truncated protein | [17] | |
| *LamB3^IAP^* | C3H | Intron/exon junction (5’), - | Transcript contains internal IAP sequence, No mRNA or protein expression | [18] | |
| *Mgrn1^md^* | C3H/HeJ | Intron 11, + | Reduced mRNA levels, aberrantly sized transcripts | [19,20] | |
| *Mgrn1^md-2J^* | C3H/HeJ | Exon 12, + | Reduced mRNA levels, aberrantly sized transcripts | [19,20] | |
| *Mgrn1^md-5J^* | C3H/HeJ | Intron 2, + | Not characterized | [19,20] | |
| *Pitpna^vb^* | DBA/2J | Intron 4, ? | Transcript contains internal IAP sequence, Reduced mRNA & protein | [21] | |
| *Reln^rl-Alb2^* | C3H/HeJ | Exon 36, - | Exon skipping, Reduced mRNA levels | [22] | |
| *Spna1^Dem^* | CcS3/Dem | Intron 10/exon 11 junction, ? | Exon skipping, Reduced protein expression, Reduced α-/β-spectrin dimer and tetramer stability | [23] | |
| *Tyr^cm1OR^*^†^ | C3Hf/R1 | Upstream, - | IAP does not form 5’-end of transcript, Reduced mRNA expression | [24] | |
| *Usp14^axJ^* | CBA | Intron 5, ? | Transcript contains internal IAP sequence^‡^, Reduced mRNA levels,  Truncated protein | [25] | |
| *Wnt9b^clf1^* | A/WySn | Downstream, + | Not characterized | [26] | |
|  |  |  |  |  | |
| **IAP Strain variants:** | | | | | |
| *Adamts13Short isoform* | multiple | Intron 23, + | Transcript and truncated protein contains internal IAP sequence, Enzyme function unaffected, transcripts terminate in IAP | | [27] |
| *Cabp5^IAP*^* | C57BL/6 | Intron 6, - | IAP forms 5’end of alternative transcript, Regulation by methylation, IAP causes premature mRNA truncation, Various truncated proteins | | [28] |
| *Ccrn4l* | multiple | Intron 1, + | IAP forms 5’-end of transcript^¥^, Increased hepatic expression in aging mice due to gradual demethylation of IAP | | [29,30] |
| *Pcdhα v8* | multiple | Exon v8, - | IAP forms 5’-end of transcript, Reduced mRNA in developing brain due to increased methylation, Increased mRNA in neuroblastoma lines | | [31] |
| *Psg23^LTR^* | multiple | Downstream^#^, + | Transcripts terminate in IAP | | [32] |
|  |  |  |  | |  |

**Supplementary Table 1: continued**

| **Etn/MusD insertions:** | | | | |
| --- | --- | --- | --- | --- |
| **Mutation** | **Strain** | **Location &**  **Orientation** | **Mutational mechanism(s)** | **Refs** |
|  |  |  |  |  |
| *Adcy1^brl^* | ICR | Intron 14, + | Transcripts contain ETn sequence, Transcripts terminate within the ETn | [33,34] |
| *Cacng2^stg^* | A/J | Intron 2, + | Transcripts contain ETn sequence^‡^, Probable termination within ETn | [35,36] |
| *Cacng2^stg-wag^* | MRL/MpJ | Intron 1, + | Transcripts contain ETn sequence^‡^, Probable termination within ETn | [35,36] |
| *Cacng2^stg-3J^* | BALB/cJ | Intron 2, + | Transcripts contain internal ETn sequence^‡^ | [35,36] |
| *Clcn1^adr^* | A2G | Intron 12, + | Transcripts contain ETn sequence, Termination within ETn | [37] |
| *Edar^Dl-slk^* | random-bred? | unknown | Transcripts contain ETn sequence, Termination within ETn | [38] |
| *Fas^lpr^* | MRL/Mp | Intron 2, + | Transcripts contain ETn sequence^‡^, Termination within ETn | [39-41] |
| *Fbxw4^Dac^* | SM/Ckc | 10kb 5’ of exon 1, - | No evident difference in size or abundance of transcript, MusD insertion | [42,43] |
| *Fbxw4^Dac-2J^* | MRL/MpJ | Intron, ? | Small amounts of normal transcript, MusD insertion | [42,43] |
| *Fign^fi^* | random-bred | Intron 2, + | Transcripts contain ETn sequence^‡^, Probable termination within ETn | [44] |
| *Foxn1^nu-Bc^* | SELH/Bc | Intron, - | Transcripts contain internal ETn sequence | [45] |
| *Gli3^pdn^* | Jcl:ICR | Intron 3, + | 5 alternatively spliced transcripts contain ETn sequence^‡^, 3 terminate within ETn, 2 terminate as wild-type | [46] |
| *Hk1^dea^* | A/J | Intron 4, ? | Probable aberrant splicing of ETn sequences into mRNA, Decreased hexokinase activity | [47] |
| *Lep^0b-2J^* | SM/Ckc-Fbxw4^Dac^ | Intron 1,^♦^ + | Transcripts contain ETn sequence, Probable termination within ETn, Loss of leptin expression. | [48] |
| *Mip^Cat-Fr^* | A/J | Intron 3 ^♦^ + | Transcripts contain ETn sequence, Termination within ETn, LTR sequence translated to protein domain | [49,50] |
| *Muted^mu^* | CHMU/Le | Intron 3,^♦^ + | Transcripts contain internal ETn sequence^‡^ | [51] |
| *Ttc7^fsn^* | A/J | Intron 14, + | Transcripts contain internal ETn sequence | [52] |
| *T^Wis^* | A/J | splice donor of exon 7, - | 8 alternatively spliced transcripts, 4 contain ETn sequence, 1 terminates within ETn, 7 terminate as wild-type, 5 have exon skipping | [53,54] |
| *Tyr^c-Bc3^* | SELH/Bc | Exon 1, - | Transcripts probably contain ETn seq., Probable termination in ETn | [45] |
|  |  |  |  |  |
| **ETn strain variants:** | | | | |
| *Dysf ^prmd^* | A/J | Intron 4,^♦^ + | Aberrant splicing, No detectable dysferlin protein | [55] |
| *Zhx2^b^* | BALB/cJ | Intron 1, + | Transcripts contain ETn sequence^‡^, Termination within ETn | [56] |
| *H2^Q6/Q8^* | C57BL/6 | Intron 3,^# ♦^ + | Larger transcripts, Altered MHC gene expression | [57] |
| *wiz* | C57BL/6 | Exon 6, - | Decreased expression of the long *wiz* isoform | [58] |
|  |  |  |  |  |
| **Other ERV insertions:** | | | | |
| *a* | Fancy mice | VL30, Intron 1, - | Smaller agouti message and levels 8 fold lower than in wild-type. | [59] |
| *Abcb1a^mds^* | CF1  Strain variant | MuLV^#^, Intron 22, - | Transcripts contain viral sequence, Exon skipping, Disruption of gene function | [60] |
| *Hr^hr^* | HRS | MuLV, Intron 6, + | Transcripts contain viral sequence^‡^, Probable termination within LTR | [61,62] |
| *Myo5a^d^* | DBA/2J | MuLV, Intron, + | Shortened and abnormally spliced transcripts that vary among tissues | [63] |
| *Nox3^het^* | GL/Le | Intron 12, ? | Transcripts show aberrant splicing from *Nox3* into retroviral element | [64] |
| *Npc1^m1N^* | BALB/c | MaLR | Transcripts contain MaLR sequence, decreased expression, premature truncation | [65] |
| *Pdcd8^Hq^* | CF1 | MuLV, Intron 1, + | 80% decrease in transcipt and protein levels, Aberrant splicing | [66] |
| *Pde6b^rd1^* | multiple | MuLV, Intron 1, - | Aberrant splicing containing viral sequence | [67] |
|  |  |  |  |  |

*	Variable phenotype or expression (metastable epiallele)

^#^	Solitary LTR

^†^	Somatic mosaic

^‡^	Alternative splicing results in some wild-type transcripts

^¥^	Ectopic expression of IAP-driven transcript

^b^	- = antisense, + = sense, ? = orientation unknown

^♦^	MusD or Etn

References:

1.	Argeson AC, Nelson KK, Siracusa LD (1996) Molecular basis of the pleiotropic phenotype of mice carrying the *hypervariable yellow* (A*^hvy^*) mutation at the *agouti* locus. Genetics 142: 557-567.

2.	Michaud EJ, van Vugt MJ, Bultman SJ, Sweet HO, Davisson MT, et al. (1994) Differential expression of a new dominant agouti allele (Aiapy) is correlated with methylation state and is influenced by parental lineage. Genes Dev 8: 1463-1472.

3.	Duhl DM, Vrieling H, Miller KA, Wolff GL, Barsh GS (1994) Neomorphic *agouti* mutations in obese yellow mice. Nat Genet 8: 59-65.

4.	Morgan HD, Sutherland HG, Martin DI, Whitelaw E (1999) Epigenetic inheritance at the agouti locus in the mouse. Nat Genet 23: 314-318.

5.	Kantheti P, Diaz ME, Peden AE, Seong EE, Dolan DF, et al. (2003) Genetic and phenotypic analysis of the mouse mutant *mh^2J^*, an *Ap3d* allele caused by IAP element insertion. Mamm Genome 14: 157-167.

6.	Bomar JM, Benke PJ, Slattery EL, Puttagunta R, Taylor LP, et al. (2003) Mutations in a novel gene encoding a CRAL-TRIO domain cause human Cayman ataxia and ataxia/dystonia in the jittery mouse. Nat Genet 35: 264-269.

7.	Gunn TM, Inui T, Kitada K, Ito S, Wakamatsu K, et al. (2001) Molecular and phenotypic analysis of *Attractin* mutant mice. Genetics 158: 1683-1695.

8.	Vasicek TJ, Zeng L, Guan XJ, Zhang T, Costantini F, et al. (1997) Two dominant mutations in the mouse *fused* gene are the result of transposon insertions. Genetics 147: 777-786.

9.	Rakyan VK, Chong S, Champ ME, Cuthbert PC, Morgan HD, et al. (2003) Transgenerational inheritance of epigenetic states at the murine *Axin^Fu^* allele occurs after maternal and paternal transmission. Proc Natl Acad Sci USA 100: 2538-2543.

10.	Ware ML, Fox JW, Gonzalez JL, Davis NM, Lambert dR, et al. (1997) Aberrant splicing of a mouse disabled homolog, mdab1, in the scrambler mouse. Neuron 19: 239-249.

11.	Sheldon M, Rice DS, D'Arcangelo G, Yoneshima H, Nakajima K, et al. (1997) *Scrambler* and *yotari* disrupt the *disabled* gene and produce a *reeler*-like phenotype in mice. Nature 389: 730-733.

12.	Johnson KR, Cook SA, Erway LC, Matthews AN, Sanford LP, et al. (1999) Inner ear and kidney anomalies caused by IAP insertion in an intron of the *Eya1* gene in a mouse model of BOR syndrome. Hum Mol Genet 8: 645-653.

13.	Gwynn B, Lueders K, Sands MS, Birkenmeier EH (1998) Intracisternal A-particle element transposition into the murine β-glucuronidase gene correlates with loss of enzyme activity: a new model for β-glucuronidase deficiency in the C3H mouse. Mol Cell Biol 18: 6474-6481.

14.	Suzuki T, Li W, Zhang Q, Novak EK, Sviderskaya EV, et al. (2001) The gene mutated in cocoa mice, carrying a defect of organelle biogenesis, is a homologue of the human Hermansky-Pudlak syndrome-3 gene. Genomics 78: 30-37.

15.	Gardner JM, Wildenberg SC, Keiper NM, Novak EK, Rusiniak ME, et al. (1997) The mouse pale ear (*ep*) mutation is the homologue of human Hermansky-Pudlak syndrome. Proc Natl Acad Sci USA 94: 9238-9243.

16.	Zhang Q, Zhao B, Li W, Oiso N, Novak EK, et al. (2003) *Ru2* and *Ru* encode mouse orthologs of the genes mutated in human Hermansky-Pudlak syndrome types 5 and 6. Nat Genet 33: 145-153.

17.	Besse S, Allamand V, Vilquin JT, Li Z, Poirier C, et al. (2003) Spontaneous muscular dystrophy caused by a retrotransposal insertion in the mouse laminin α2 chain gene. Neuromuscul Disord 13: 216-222.

18.	Kuster JE, Guarnieri MH, Ault JG, Flaherty L, Swiatek PJ (1997) IAP insertion in the murine *LamB3* gene results in junctional epidermolysis bullosa. Mamm Genome 8: 673-681.

19.	Phan LK, Lin F, LeDuc CA, Chung WK, Leibel RL (2002) The mouse *mahoganoid* coat color mutation disrupts a novel C3HC4 RING domain protein. J Clin Invest 110: 1449-1459.

20.	He L, Lu XY, Jolly AF, Eldridge AG, Watson SJ, et al. (2003) Spongiform degeneration in mahoganoid mutant mice. Science 299: 710-712.

21.	Hamilton BA, Smith DJ, Mueller KL, Kerrebrock AW, Bronson RT, et al. (1997) The vibrator mutation causes neurodegeneration via reduced expression of PITP alpha: positional complementation cloning and extragenic suppression. Neuron 18: 711-722.

22.	Royaux I, Bernier B, Montgomery JC, Flaherty L, Goffinet AM (1997) *Reln^rl-Alb2^*, an allele of reeler isolated from a chlorambucil screen, is due to an IAP insertion with exon skipping. Genomics 42: 479-482.

23.	Wandersee NJ, Roesch AN, Hamblen NR, de Moes J, van der Valk MA, et al. (2001) Defective spectrin integrity and neonatal thrombosis in the first mouse model for severe hereditary elliptocytosis. Blood 97: 543-550.

24.	Wu M, Rinchik EM, Wilkinson E, Johnson DK (1997) Inherited somatic mosaicism caused by an intracisternal A particle insertion in the mouse tyrosinase gene. Proc Natl Acad Sci USA 94: 890-894.

25.	Wilson SM, Bhattacharyya B, Rachel RA, Coppola V, Tessarollo L, et al. (2002) Synaptic defects in *ataxia* mice result from a mutation in *Usp14*, encoding a ubiquitin-specific protease. Nat Genet 32: 420-425.

26.	Juriloff DM, Harris MJ, Dewell SL, Brown CJ, Mager DL, et al. (2005) Investigations of the genomic region that contains the *clf1* mutation, a causal gene in multifactorial cleft lip and palate in mice. Birth Defects Res 73: 103-113.

27.	Banno F, Kaminaka K, Soejima K, Kokame K, Miyata T (2004) Identification of strain-specific variants of mouse *Adamts13* gene encoding von Willebrand factor-cleaving protease. J Biol Chem 279: 30896-30903.

28.	Druker R, Bruxner TJ, Lehrbach NJ, Whitelaw E (2004) Complex patterns of transcription at the insertion site of a retrotransposon in the mouse. Nucleic Acids Res 32: 5800-5808.

29.	Barbot W, Dupressoir A, Lazar V, Heidmann T (2002) Epigenetic regulation of an IAP retrotransposon in the aging mouse: progressive demethylation and de-silencing of the element by its repetitive induction. Nucleic Acids Res 30: 2365-2373.

30.	Puech A, Dupressoir A, Loireau MP, Mattei MG, Heidmann T (1997) Characterization of two age-induced intracisternal A-particle-related transcripts in the mouse liver. Transcriptional read-through into an open reading frame with similarities to the yeast ccr4 transcription factor. J Biol Chem 272: 5995-6003.

31.	Sugino H, Toyama T, Taguchi Y, Esumi S, Miyazaki M, et al. (2004) Negative and positive effects of an IAP-LTR on nearby *Pcda*α gene expression in the central nervous system and neuroblastoma cell lines. Gene 337: 91-103.

32.	Ball M, McLellan A, Collins B, Coadwell J, Stewart F, et al. (2004) An abundant placental transcript containing an IAP-LTR is allelic to mouse pregnancy-specific glycoprotein 23 (*Psg23*): cloning and genetic analysis. Gene 325: 103-113.

33.	Abdel-Majid RM, Leong WL, Schalkwyk LC, Smallman DS, Wong ST, et al. (1998) Loss of adenylyl cyclase I activity disrupts patterning of mouse somatosensory cortex. Nat Genet 19: 289-291.

34.	Leong WL, Dobson MJ, Logsdon JM, Jr., Abdel-Majid RM, Schalkwyk LC, et al. (2000) ETn insertion in the mouse Adcy1 gene: transcriptional and phylogenetic analyses. Mamm Genome 11: 97-103.

35.	Letts VA, Felix R, Biddlecome GH, Arikkath J, Mahaffey CL, et al. (1998) The mouse stargazer gene encodes a neuronal Ca^2+^-channel γ subunit. Nat Genet 19: 340-347.

36.	Letts VA, Kang MG, Mahaffey CL, Beyer B, Tenbrink H, et al. (2003) Phenotypic heterogeneity in the stargazin allelic series. Mamm Genome 14: 506-513.

37.	Schnulle V, Antropova O, Gronemeier M, Wedemeyer N, Jockusch H, et al. (1997) The mouse Clc1/myotonia gene: ETn insertion, a variable AATC repeat, and PCR diagnosis of alleles. Mamm Genome 8: 718-725.

38.	Headon DJ, Overbeek PA (1999) Involvement of a novel Tnf receptor homologue in hair follicle induction. Nat Genet 22: 370-374.

39.	Wu J, Zhou T, He J, Mountz JD (1993) Autoimmune disease in mice due to integration of an endogenous retrovirus in an apoptosis gene. J Exp Med 178: 461-468.

40.	Adachi M, Watanabe-Fukunaga R, Nagata S (1993) Aberrant transcription caused by the insertion of an early transposable element in an intron of the Fas antigen gene of *lpr* mice. Proc Natl Acad Sci USA 90: 1756-1760.

41.	Chu JL, Drappa J, Parnassa A, Elkon KB (1993) The defect in *Fas* mRNA expression in MRL/*lpr* mice is associated with insertion of the retrotransposon, ETn. J Exp Med 178: 723-730.

42.	Sidow A, Bulotsky MS, Kerrebrock AW, Birren BW, Altshuler D, et al. (1999) A novel member of the F-box/WD40 gene family, encoding dactylin, is disrupted in the mouse dactylaplasia mutant. Nat Genet 23: 104-107.

43.	Kano,H., Kurahashi,H., and Toda,T. (2004). The dactylaplasia phenotype is suppressed by the silencing of its mutational retrotransposon. The American Society of Human Genetics Annual Meeting, Toronto, Canada (Abstract).

44.	Cox GA, Mahaffey CL, Nystuen A, Letts VA, Frankel WN (2000) The mouse fidgetin gene defines a new role for AAA family proteins in mammalian development. Nat Genet 26: 198-202.

45.	Hofmann M, Harris M, Juriloff D, Boehm T (1998) Spontaneous mutations in SELH/Bc mice due to insertions of early transposons: molecular characterization of null alleles at the nude and albino loci. Genomics 52: 107-109.

46.	Thien H, Ruther U (1999) The mouse mutation *Pdn* (Polydactyly Nagoya) is caused by the integration of a retrotransposon into the *Gli3* gene. Mamm Genome 10: 205-209.

47.	Peters LL, Lane PW, Andersen SG, Gwynn B, Barker JE, et al. (2001) Downeast anemia (*dea*), a new mouse model of severe nonspherocytic hemolytic anemia caused by hexokinase (HK_1_) deficiency. Blood Cells Mol Dis 27: 850-860.

48.	Moon BC, Friedman JM (1997) The molecular basis of the obese mutation in ob^2J^ mice. Genomics 42: 152-156.

49.	Shiels A, Bassnett S (1996) Mutations in the founder of the *MIP* gene family underlie cataract development in the mouse. Nat Genet 12: 212-215.

50.	Shiels A, Mackay D, Bassnett S, Al Ghoul K, Kuszak J (2000) Disruption of lens fiber cell architecture in mice expressing a chimeric AQP0-LTR protein. FASEB J 14: 2207-2212.

51.	Zhang Q, Li W, Novak EK, Karim A, Mishra VS, et al. (2002) The gene for the muted (*mu*) mouse, a model for Hermansky-Pudlak syndrome, defines a novel protein which regulates vesicle trafficking. Hum Mol Genet 11: 697-706.

52.	White RA, McNulty SG, Nsumu NN, Boydston LA, Brewer BP, et al. (2005) Positional cloning of the *Ttc7* gene required for normal iron homeostasis and mutated in *hea* and *fsn* anemia mice. Genomics 85: 330-337.

53.	Herrmann BG, Labeit S, Poustka A, King TR, Lehrach H (1990) Cloning of the T gene required in mesoderm formation in the mouse. Nature 343: 617-622.

54.	Goldin SN, Papaioannou VE (2003) Unusual misregulation of RNA splicing caused by insertion of a transposable element into the *T* (*Brachyury*) locus. BMC Genomics 4: 14.

55.	Ho M, Post CM, Donahue LR, Lidov HG, Bronson RT, et al. (2004) Disruption of muscle membrane and phenotype divergence in two novel mouse models of dysferlin deficiency. Hum Mol Genet 13: 1999-2010.

56.	Perincheri S, Dingle RW, Peterson ML, Spear BT (2005) Hereditary persistence of α-fetoprotein and *H19* expression in liver of BALB/cJ mice is due to a retrovirus insertion in the *Zhx2* gene. Proc Natl Acad Sci USA 102: 396-401.

57.	Elliott E, Rathbun D, Ramsingh A, Garberi J, Flaherty L (1989) Genetics and expression of the Q6 and Q8 genes. An LTR-like sequence in the 3' untranslated region. Immunogenetics 29: 371-379.

58.	Baust C, Baillie GJ, Mager DL (2002) Insertional polymorphisms of ETn retrotransposons include a disruption of the *wiz* gene in C57BL/6 mice. Mamm Genome 13: 423-428.

59.	Bultman SJ, Klebig ML, Michaud EJ, Sweet HO, Davisson MT, et al. (1994) Molecular analysis of reverse mutations from nonagouti (a) to black-and-tan (a(t)) and white-bellied agouti (Aw) reveals alternative forms of agouti transcripts. Genes Dev 8: 481-490.

60.	Jun K, Lee SB, Shin HS (2000) Insertion of a retroviral solo long terminal repeat in mdr-3 locus disrupts mRNA splicing in mice. Mamm Genome 11: 843-848.

61.	Stoye JP, Fenner S, Greenoak GE, Moran C, Coffin JM (1988) Role of endogenous retroviruses as mutagens: the hairless mutation of mice. Cell 54: 383-391.

62.	Cachon-Gonzalez MB, Fenner S, Coffin JM, Moran C, Best S, et al. (1994) Structure and expression of the hairless gene of mice. Proc Natl Acad Sci USA 91: 7717-7721.

63.	Jenkins NA, Copeland NG, Taylor BA, Lee BK (1981) Dilute (*d*) coat colour mutation of DBA/2J mice is associated with the site of integration of an ecotropic MuLV genome. Nature 293: 370-374.

64.	Paffenholz R, Bergstrom RA, Pasutto F, Wabnitz P, Munroe RJ, et al. (2004) Vestibular defects in head-tilt mice result from mutations in *Nox3*, encoding an NADPH oxidase. Genes Dev 18: 486-491.

65.	Loftus SK, Morris JA, Carstea ED, Gu JZ, Cummings C, et al. (1997) Murine model of Niemann-Pick C disease: mutation in a cholesterol homeostasis gene. Science 277: 232-235.

66.	Klein JA, Longo-Guess CM, Rossmann MP, Seburn KL, Hurd RE, et al. (2002) The harlequin mouse mutation downregulates apoptosis-inducing factor. Nature 419: 367-374.

67.	Bowes C, Li T, Frankel WN, Danciger M, Coffin JM, et al. (1993) Localization of a retroviral element within the *rd* gene coding for the β subunit of cGMP phosphodiesterase. Proc Natl Acad Sci USA 90: 2955-2959.
